# Progress in Psychiatry

**Published:** 1902-01-11

**Authors:** 


### PROGRESS IN PSYCHIATRY.

**Melancholia and the Vesanic Psychoses.**—The condition of melancholia comprises a number of cerebral and bodily states, of which the following are the chief:—A depression of spirits or of the general feeling of well-being, an emotion of fear, a feeling of fatigue with inability for exertion, a brooding upon anxious thoughts and fears, and a marked disturbance of the digestive and assimilative processes of health. The combination of these elements and their persistence and development to such an extent as to endanger the mental life of the individual constitutes melancholia. "A melancholiac," says Griesinger, "is habitually tired, and if compelled to undergo great exertion will certainly become worse, and probably pass into mania or dementia." An element of gloom or fear pervades the thoughts and feelings, and this even while a fair degree of mental lucidity is maintained. The physical signs are dryness and coldness of the skin and extremities; loss of appetite, with gastric disturbances such as fermentation and heartburn; insomnia and a dull headache, with more or less tenderness of the scalp; hard pulse, with high radial tension; constipation and ischuria; and general muscular weakness with slowness and difficulty in executing fine movements. The melancholiac loses interest in duties and pleasures alike, he forsakes his habitual occupations, the general sensorial and kinæsthetic life tends to diminish in activity, and painful thoughts and anxious fears of a vague character besiege his mind. According to the acuteness of its manifestations melancholia may be regarded as including three grades, as follows:—

*a.* Simple depression of spirits.
*b.* Simple or sub-acute melancholia.
*c.* Acute melancholia in its various forms.

As a rule, melancholia "occurs mostly in timid, reserved, timorous, and scrupulous individuals (Regis [1])." They are generally of good physique and of more than average intelligence and sensibility. Melancholia is more frequent amongst women than men, as their habits and life encourage conditions favourable to the development of melancholia. It occurs without premonitions as a rule, and chiefly affects people who are hereditarily predisposed to neuroses and psychoses. It may occur at any period of life, from adolescence to old age. Apart from hereditary predisposition, the most frequent causes are business worry and anxiety, great financial losses, grief from deaths or other domestic calamities, and such debilitating processes as starvation, over-fatigue, puerperal stresses, and post-febrile exhaustion. Gastro-intestinal disorder with auto-intoxication may be a more frequent cause than is usually believed, especially since recent researches by Magnan, Kraepelin,[2] Cololian,[3] Marinesco, Herter,[4] and others have shown that auto-intoxication is a frequent cause of mental confusion and stupor, status epilepticus, acute (uræmic) delirium, and so-called katatomic melancholia.[5] Lange, of Copenhagen, showed in a memoir published in 1886 that a condition of simple depression of spirits may occur in some persons periodically or at regular intervals and may continue throughout life without passing into actual melancholic or other forms of insanity. A translation of Dr. Lange's monograph was made by Dr. Kurella and published at Leipzig in 1887. A classical instance of this form of depression of spirits was Carlyle.

[1] Regis, Mental Medicine, p. 170. [2] Kraepelin's Psychiatrie, 1900. [3] Archives de Neurologie, 1899. [4] Journal of Nervous and Mental Diseases, 1899. [5] Regis and Lalanne in Archives de Neurologie, October, 1901.

